# Studying mutation rate evolution in primates—a need for systematic comparison of computational pipelines

**DOI:** 10.1093/gigascience/giab072

**Published:** 2021-10-21

**Authors:** Lucie A Bergeron, Søren Besenbacher, Mikkel H Schierup, Guojie Zhang

**Affiliations:** Section for Ecology and Evolution, Department of Biology, University of Copenhagen, 2100, Copenhagen, Denmark; Department of Molecular Medicine, Aarhus University, 8200, Aarhus, Denmark; Bioinformatics Research Centre, Aarhus University, 8000, Aarhus, Denmark; Section for Ecology and Evolution, Department of Biology, University of Copenhagen, 2100, Copenhagen, Denmark; BGI-Shenzhen, Shenzhen, Guangdong 518083, China; State Key Laboratory of Genetic Resources and Evolution, Kunming Institute of Zoology, Chinese Academy of Sciences, Kunming 650223, China; Center for Excellence in Animal Evolution and Genetics, Chinese Academy of Sciences, Kunming 650223, China

**Keywords:** germline mutation rate, NGS sequencing, computational pipeline

## Abstract

The lack of consensus methods to estimate germline mutation rates from pedigrees has led to substantial differences in computational pipelines in the published literature. Here, we answer Susanne Pfeifer's opinion piece discussing the pipeline choices of our recent article estimating the germline mutation rate of rhesus macaques (*Macaca mulatta*). We acknowledge the differences between the method that we applied and the one preferred by Pfeifer. Yet, we advocate for full transparency and justification of choices as long as rigorous comparison of pipelines remains absent because it is the only way to conclude on best practices for the field.

## Introduction

We agree with the comments from Susanne Pfeifer [1] that choice of computational pipeline is important for germline mutation rate estimation and there is no agreement yet on a consensus pipeline. Several studies have published mutation rate estimations in different species, using different experimental designs and computational approaches (see Table 1 for key settings used in the articles cited by Pfeifer). To make the results comparable, it is essential for the community to agree upon some of the critical criteria in the pipeline for mutation rate estimation. Thus, we organized a Mutationathon consortium involving active research groups in this field to compare different pipelines on the same pedigree data and measure the effect of some criteria in the pipeline. The results of this consortium effort also emphasize the effect of different bioinformatic pipelines on estimated rates. We provided some guidelines on the different steps of the analysis, yet agreed that many criteria should be chosen according to the data available (e.g., size of the pedigree, sequencing coverage). The outcome of this comparison and our discussion are now available as a preprint [2]. Until scientifically validated best practices are established for all steps of the analysis, it is of great importance to make all data and scripts available together with the choices of key settings in a way that the studies can be reproduced and in order to make it easy for other groups to reanalyze the data with different settings. This is what we have already done in our study.

**Table 1:** Comparison of key criteria used in our study and in 7 other studies on non-human primates cited by Pfeifer [1].

Criterion	Venn et al. [3]	Pfeifer [4]	Tatsumoto et al. [5]	Thomas et al. [6]	Besenbacher et al. [7]	Wang et al. [8]	Wu et al. [9]	Our study [10]
Type of library	PCR free	PCR	PCR free	PCR free	PCR free	PCR free	PCR free	PCR
BQSR	No	Yes	Yes	No	Yes	No	Yes	No
Variant caller	Cortex	GATK	GATK	GATK	GATK	GATK	GATK	GATK
GQ	20	20	PL < 200, heterozygous; PL < 100, homozygous	20	65	70	40	60
Simulation to estimate FNR	Yes	Yes	No	No	No	No	Yes	No

FNR: false-negative rate; GQ: genotype quality; PL: Phred-scaled likelihood.

Despite Pfeifer acknowledging that no consensus has been established on the germline mutation rate estimation, she argued that some criteria used in our pipeline were not the best choice. Specifically, these criteria are the *de novo* variant filtering, manual curation, and estimation of the false-negative rate (FNR). The majority of these points have been extensively discussed in our original publication and in the peer review process available with the published article. Below, we provide a point-by-point discussion for each of the questions raised by Pfeifer.

### Point-by-point answer

In her commentary, Pfeifer argues that there is a difference of 32.8% between the per generation rate we estimated and the one estimated by Wang and collaborators [8] caused by biological, experimental, and methodological factors. As we discussed in our article, the difference is only 5% between the 2 studies if we compare the estimated yearly mutation rate, taking into account the parental age effect because the age of reproduction differs between the studies. While we acknowledge that the bioinformatic pipeline and experimental designs might also affect the results, and explain some of the remaining differences between the independently estimated rates, we do not agree that a 5% difference constitutes a major discrepancy. Pfeifer also argued that the sequencing technology difference would also introduce difference in the results. Nevertheless, many published benchmark studies have shown that the overall genotyping error rate, the individual Phred base quality scores of the primary reads, the uniformity of coverage, and GC coverage are comparable between BGISEQ-500 and Illumina HiSeq [see the most recent study in 11]. Yet, we are not aware of any studies comparing the effect of the type of libraries or sequencing technologies on final estimated rates.

Another concern was the insufficient justification for the genotype quality (GQ) threshold that we used. We agree that the GQ threshold is one of the most difficult filters to set up because it greatly affects the rate. Indeed, this is one of the conclusions of our recent comparative work [2]. Many studies (e.g., 4 of the 7 studies cited by Pfeifer, see Table 1) used a GQ filter of >20. Even if GATK refers to GQ 20 as “widely accepted as a good threshold for genotype accuracy, indicating that there is a 99% chance that the genotype in question is correct" (see article “Genotype Refinement workflow for germline short variants" on GATK documentation [12]), this is only for general variant calling because a few hundred genotype errors genome-wide does not count very much in comparison to millions of real variant sites. However, reducing these errors is highly important when trying to detect ∼100 *de novo* mutations. Moreover, in a sequence data set with low coverage, a variant with GQ 20 will correspond to a variant with average depth and ∼50–50 allele balance. But in a high-coverage data set, it is only unusual variants that fail to get a GQ of >20. Such variants must either have much lower than average depth or have a very uneven allele balance—and in neither case would we trust them for *de novo* mutation calling. We agree with the suggestion that having a standardized way of choosing the GQ filter could improve our ability to compare between studies. However, there is yet no consensus on how to do so, and comparing the methods is beyond the scope of our project. Therefore, we used a similar exploration method to those previously published [7]. This approach chooses the GQ threshold by calculating a mutation rate from the number of candidate *de novo* mutations divided by the estimated callability for different GQ thresholds and then chooses the lowest value of GQ where the value does not decrease, suggesting that the number of FPs is low. For full transparency, we reported rates for all tested GQ values in our article.

Moreover, Pfeifer claims that the reference genotype quality (RGQ) should be used rather than genotype quality (GQ) to calculate the callability. Figure 2 of Pfeifer's commentary [1] presents the difference between the RGQ and GQ values of 1 million invariant sites called in one of our macaque trios. Yet, complete information on how these sites were genotyped is lacking (i.e., which version of GATK was used with which commands and options). However, RGQ is not a parameter in our variant calling output. The RGQ was a parameter of GATK 3.4 (GenotypeGVCFs -allSites option). Our pipeline was based on GATK 4.0.7.0, in which “-allSites" is no longer an option for GenotypeGVCFs. Therefore, none of our files presented RGQ values in their format field, neither in the initial variant calling per sample with HapplotypeCaller nor in the final files with either all variant sites or all single positions, even the nonvariant sites. For this reason, we think it is important to report which version of GATK was used and the scripts with the different commands used to compare studies. All our scripts are available and our pipeline with the different commands used is described in Supplementary Fig. S8. We agree that there is a distinction on the calculation of GQ for variant and non-variant sites. In both cases, GQ is the difference between the most likely PL and the second most likely. This latter cannot be estimated from a conditional probability in the same way for non-variant sites as it is for variant sites, but this should not be a problem for the estimation of the callable genome (see the articles “Calculation of PL and GQ by HaplotypeCaller and GenotypeGVCFs" and “HaplotypeCaller Reference Confidence Model (GVCF mode)" on GATK documentation)[12].

**Figure 1:**
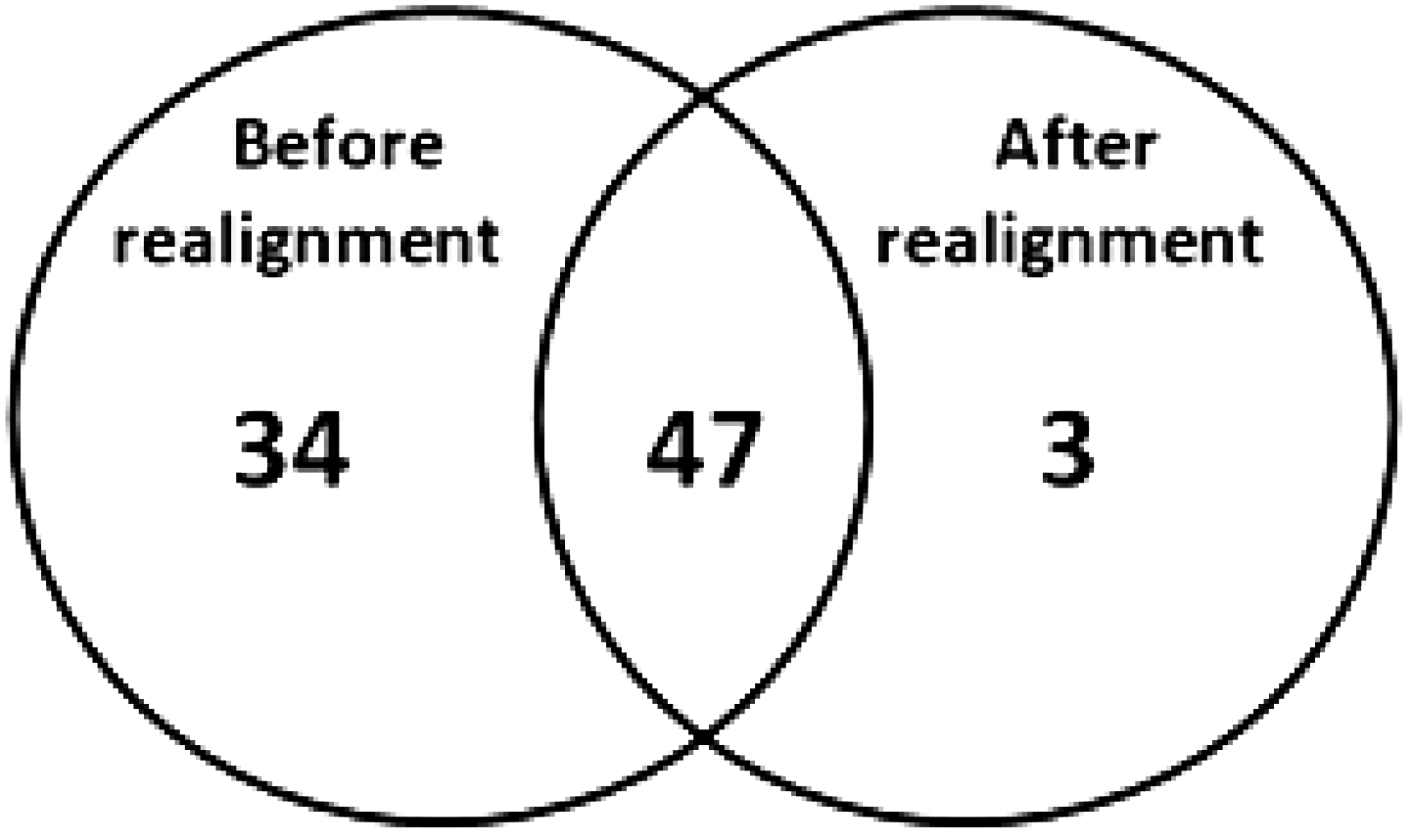
The number of potential false-positive candidates found by the manual curation method from the Bams before realignment and after realignment.

Pfeifer also disagrees with our method to correct for false-positive calls (FPs), stating that the manual curation should be done on a final set of realigned haplotypes rather than the initial genotype calls. We do not think that there is any scientific consensus on whether to manually curate on BAMs from primary genotype calls or haplotype-realigned BAMs. That is the reason why we did both in our article, along with some PCR experiments and resequencing. We kept the manual curation before realignment, which included 96% of the potential FPs (81 of 84) while the manual curation after realignment would have only included 60% of potential FPs (50 of 84) (Figure 1). One argument for this is that realignment is only performed on complex regions in the first place and thus, *de novo* mutation calls in such regions are associated with much more uncertainty. Consistent with our choice, our PCR validation with Sanger sequencing confirmed the false-positive nature of one of the 34 FPs detected by the manual curation before realignment, while this candidate incorrectly appeared as a true-positive call with the curation after realignment. However, we agree that choosing one or the other strategy affects the estimated rate. In our case the confidence levels between rates estimated in these 2 ways overlap, but we still found it important to discuss the point in our article and provide the information needed to calculate the rate based on curation after realignment.

Finally, the preferred method of Pfeifer to estimate the FNR is by simulation of mutation, again reflecting the lack of consensus on methodology. These simulations were used in some studies to estimate FNR, as in 3 of the 7 studies mentioned by Pfeifer (see Table 1). Other studies chose a different methodology, similar to our study, and did not perform simulation. While we agree that the simulation could incorporate the risk of mismapping errors when a mutation is present, it could also change the mapping quality, GQ, and depth at many positions. Therefore, to correctly assess the aligner performance with the simulation method a new callability should be assessed. As we estimated the callability from the BP_RESOLUTION files using the same filters as for the candidate site we estimated an FNR for this callable genome using only the site filters and allelic balance filter.

## Conclusion

In her commentary, Pfeifer suggested her personal favored criteria on the pipeline choices, which reflects the lack of a consensus in the field on the pipeline in calculating mutation rate. We agree that more effort is needed to comprehensively compare different methods. Such an effort has been started and we now have an idea of how some filters can reduce the occurrence of FPs and affect the final rate [2]. Yet, the same type of comparison should be made to explore the effect of the different parameters that Pfeifer mentioned here, such as sequencing technologies, different methods to correct FPs, and to estimate the FNRs on the final estimated rate. Because the “ground truth" is hard to access, the only way to do so is by systematic comparison, which is beyond the scope of our original article.

## Editor's Note

Several recent studies by different groups present data on mutation rate estimation for primates derived from pedigree sequencing. Within this active and new field, a range of analysis methods are being employed. As the review process of Bergeron et al. [10] has shown, there are different views regarding the choice of particular methods and pipelines. Following up on the review process, this article is part of an exchange of opinions between one of the reviewers [1] and the authors (this commentary), in the spirit of contributing to the development of consensus in this rapidly developing area of research.

## Abbreviations

BQSR: Base Quality Score Recalibration; FNR: false-negative rate; FP: false-positive; GATK: Genome Analysis Toolkit; GQ: genotype quality; PL: Phred-scaled likelihoods; RGQ: reference genotype quality.

## Data Availability

Not applicable.

## Competing Interests

The authors declare that they have no competing interests.

## Funding

This work was supported by a Carlsberg Foundation Grant to G.Z. (CF16-0663).

## Authors' Contributions

All authors wrote the manuscript.
